# MicroRNA Function in the Profibrogenic Interplay upon Chronic Liver Disease

**DOI:** 10.3390/ijms15069360

**Published:** 2014-05-27

**Authors:** Jia Huang, Xiaojie Yu, Jochen W. U. Fries, Li’ang Zhang, Margarete Odenthal

**Affiliations:** 1 Institute for Pathology, University Hospital of Cologne, Cologne 50924, Germany; E-Mails: jia.huang@uk-koeln.de (J.H.); xiaojie.yu@uk-koeln.de (X.Y.); jochen.fries@uni-koeln.de (J.W.U.F.); zhang.liang@uk-koeln.de (L.Z.); 2 Center for Molecular Medicine Cologne, University of Cologne, Cologne 50924, Germany; 3 Clinic I for Internal Medicine, University Hospital of Cologne, Cologne 50924, Germany

**Keywords:** liver fibrosis, myofibroblastic transition, TGF-β, miRNA

## Abstract

In chronic liver disease leading to fibrosis, hepatic stellate cells (HSC) differentiate into myofibroblasts. Myofibroblastic HSC have taken center stage during liver fibrogenesis, due to their remarkable synthesis of extracellular matrix proteins, their secretion of profibrogenic mediators and their contribution to hypertension, due to elevated contractility. MicroRNAs (miRNAs) are small, noncoding RNA molecules of 19–24 nucleotides in length. By either RNA interference or inhibition of translational initiation and elongation, each miRNA is able to inhibit the gene expression of a wide panel of targeted transcripts. Recently, it was shown that altered miRNA patterns after chronic liver disease highly affect the progression of fibrosis by their potential to target the expression of extracellular matrix proteins and the synthesis of mediators of profibrogenic pathways. Here, we underline the role of miRNAs in the interplay of the profibrogenic cell communication pathways upon myofibroblastic differentiation of hepatic stellate cells in the chronically injured liver.

## 1. Introduction

MicroRNAs are small, 19–24 nucleotides-long, non-coding RNA molecules. They are transcribed by RNA polymerase II as primary transcripts (pri-miRNA). Pri-miRNAs are subsequently processed by the RNase III enzyme, Drosha, forming stem-loop structured miRNA precursors (pre-miRNAs) of about 80 nucleotides. After the export of pre-miRNAs into the cytoplasm, the highly conserved RNase III enzyme, Dicer, releases the pre-miRNAs to form the mature miRNAs, which then are incorporated into the RNA-induced silencing complex (RISC). The interaction of the miRNA/RISC complex by complementary annealing of the mostly seven nucleotide-long, so-called miRNA seed sequence with the untranslated region (UTR) of mRNA, leads to the inhibition of translation [1,2,3]. In total, more than 2500 human miRNAs have been discovered [4]. Since one miRNA might target hundreds of different mRNA transcripts [2], it is suggested that miRNAs regulate more than one third of the human genes [5,6].

Due to their high impact on gene regulation, miRNAs are involved in most cellular alteration processes, such as cell proliferation, migration and differentiation. Thus, many previous reports recognized miRNAs as central players in the oncogene and tumor suppressor networks, contributing to the initiation and progression of many human malignancies [7,8]. Furthermore, miRNA-mediated gene regulation is also an important feature in acute and chronic inflammatory diseases [9,10,11,12]. Recent studies on chronic liver diseases of different etiologies revealed a prominent dysregulation of many miRNAs, leading to an altered gene expression profile and progression of liver fibrosis, previously summarized by references [13,14,15,16,17,18]). In the present short review, we summarize recent findings on the role of miRNAs in the pathophysiology of fibrosis after chronic liver injury.

## 2. Altered miRNA Expression upon Liver Fibrosis

Liver fibrosis has become one of the most serious problems for human health, which can lead to hepatocellular carcinoma. It is characterized by the hepatic accumulation of biomatrix as a pathophysiological response to chronic liver injuries independently of the causative noxa [19,20,21]. In addition to viral infections, such as chronic hepatitis B (HBV) and C (HCV) infections or excessive alcohol abuse, in the recent past, fatty liver disease leading to steatohepatitis is also a frequent agent of chronic liver injury and fibrosis, primarily due to altered food habits in Western countries [22].

Extracellular matrix (ECM) accumulation during liver fibrogenesis is mainly distinguished by a dramatically enhanced deposition of collagen I and collagen III. Especially TGF-β triggers the interstitial ECM accumulation by an induced synthesis, but also by a decreased ECM turn-over after repression of matrix-metalloproteinases [19,20,21]. In addition, the function of TGF-β as a central mediator of fibrosis is strongly manifested by its evoked stimulation of profibrogenic growth factor profiles, including TGF-β itself, the platelet-derived growth factors (PDGF), connective tissue growth factor (CTGF), endothelin (ET) and many others [23].

During liver fibrogenesis caused by different etiologies, an altered miRNA expression was observed [24,25,26,27]. miR-122 is the most abundant miRNA in normal liver, highly enriched in the liver parenchyma, accounting for more than 70% of the total miRNA population in hepatocytes [28,29]. However, the progression of fibrosis after chronic liver disease, such as chronic HCV infection or non-alcoholic liver disease, is accompanied by a predominant decrease of miR-122 [24,26,30]. These reduced miR-122 levels after fibrosis are suggested to be based on hepatocyte injury followed by miR-122 release into the blood stream, on one hand, and by transcriptional repression, due to the loss of liver-specific transcription factors, such as HNF1a and HNF4a, controlling miR-122 gene expression [31], on the other. Of special interest is the downregulation of miR-122 after chronic HCV infection, because miR-122 was shown to be involved in HCV replication. miR-122 triggers HCV replication by interaction with the 5´UTR of HCV RNA genome, resulting in the high stability of the HCV RNA [29,32,33]. These data impose miR-122 as a first target of a novel therapeutic strategy to treat chronic HCV infection [34]. Indeed, a recent clinical trial has demonstrated the successful application of miR-122 antagonists for HCV repression [35].

Furthermore, many other miRNAs are downregulated after liver fibrosis. Hence, a predominant repression of the miR-29 family members, miR-29a and miR-29b, was found in experimental fibrosis after liver intoxication or cholestasis [27]. Additionally, downregulation of miR-29c was also demonstrated in a dietary non-alcoholic steatohepatitis (NASH) mouse model [36] or in human liver fibrosis of chronically HCV-infected patients [37]. Previous findings have shown that the members of the miR-29 family act as tumor suppressor miRNAs, inhibiting the synthesis of the anti-apoptotic proteins, Bcl-2 and Mcl-1, and the DNA methyltransferases, 3A and 3B, involved in the epigenetic methylation machinery of epithelial cell types [38,39]. Most notably, the members of the miR-29 family also function as antifibrotic miRNAs, first described in cardiac fibrosis by their inhibitory role on collagen I and III, elastin and fibrillin-1 expression [40]. Inhibition of ECM synthesis and its downregulation during fibrogenesis was additionally shown in pulmonary fibrosis [41] and systemic sclerosis [42]. Furthermore, the findings of Cushing *et al*. suggested that miR-29 inhibits expression of a wide variety of fibrosis-associated genes and confirmed that numerous components of ECM are negatively regulated by miR-29, including fibrillin-1, follistatin, nidogen 1 and laminin [41].

Moreover, since the loss of miR-133 in liver fibrosis after TGF-β exposure causes prominent enhancement of collagen 1A1 and collagen 5A3 deposition, Roderburg *et al.* suggested miR-133 as a main antifibrotic miRNA [43].

On the contrary to these downregulated miRNAs, others are upregulated during fibrogenesis. Thus, miR-34a, miR-199/200 and miR-221/222 are known to be increased during liver fibrosis with different pathogenesis, such as non-alcoholic and alcoholic steatohepatitis (NASH/ASH), HCV infection or experimental fibrosis, including CCl4 intoxication and a fat diet mouse model [36,44,45,46]. The role of the miR-200 family members in liver fibrogenesis is still not completely understood. Although Murakami *et al.* have shown that miR-200a/b were positively associated with the progression of liver fibrosis in chronic hepatitis C patients [45], Sun *et al.* have reported that miR-200a is downregulated during hepatic stellate cell activation, as well as after CCl4 intoxication-based experimental fibrosis [47]. However, in agreement with Murakami *et al.*, miR-200c levels were found to be increased in the fibrotic liver after HCV-infection and NASH [48]. These opposing results may be due to diversity in disease progression or etiology.

Though the role of increased miR-34a levels upon liver fibrogenesis is not yet studied in detail, in cancer, miR-34a was shown to repress the deacetylase *SIRT1* expression, leading to a marked increase of acetylated p53, followed by elevated apoptosis [49,50]. Interestingly, miR-34a is also involved in ethanol-induced apoptosis and in hepatic remodeling by targeting matrix metalloproteinases, MMP2 and MMP9 [44]. In addition, the increase of miR-199 during fibrogenesis is suggested to contribute to hepatic remodeling by targeting the expression of ECM turnover-involved genes, like collagen *Col1A1*, the tissue inhibitor of metalloproteinase (*TIMP-1*) and the matrix metalloproteinase, *MMP13*.

## 3. Linkage of miRNA Alteration to Myofibroblastic Transition

**Figure 1:**
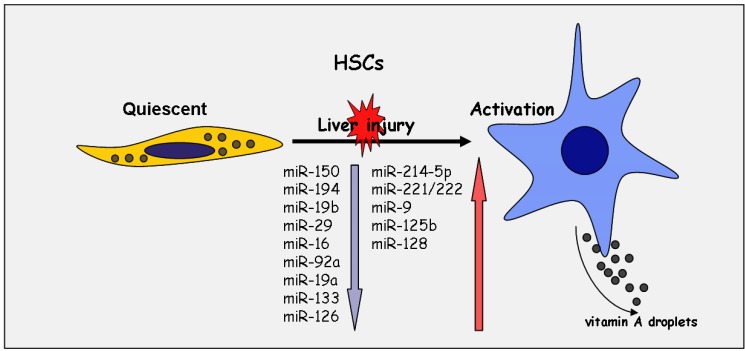
Altered expression pattern of microRNAs during myofibroblastic activation of hepatic stellate cells (HSCs).

During liver fibrogenesis, myofibroblastic cells are the central fibrotic cell type responsible for ECM accumulation. They derive from different cell types, but most of the myofibroblasts originate from hepatic stellate cells (HSCs) [51,52]. HSCs account for approximately one-third of the non-parenchymal cells and 15% of the total number of resident cells in the healthy liver [53,54]. They store around 80% of the vitamin A of the human body. However, in response to liver injury, HSCs undergo phenotypical and functional changes, including the loss of vitamin A storage, proliferation, cytoskeleton alteration and synthesis of ECM, leading to a myofibroblast phenotype with enhanced matrix deposition and contractility [51,52,55]. This transition process of HSC into a myofibroblastic cell type is accompanied by an altered growth factor profile, resulting in autocrine and paracrine profibrotic stimulation by, e.g., TGF-β, PDGF-BB, endothelin, chemokines and cytokines, and in the enhancement of fibrosis [51,52]. Importantly, previous reports have shown that several miRNA species are involved in the transdifferentiation process of quiescent HSC into a myofibroblastically activated cell type [56,57,58,59,60,61,62] (Figure 1). First, Guo *et al.* and Ji *et al.* assumed that miRNAs may be involved in the altered gene expression profile of myofibroblastic HSC [57,59].

Quiescent hepatic stellate cells (HSCs) store 80% of the body vitamin A in fat droplets. In response to chronic liver injury, HSCs undergo myofibroblastic transition. During this process, the phenotype and function of HSCs is considerably changed, including loss of vitamin A storage, proliferation, cytoskeleton alteration and synthesis of ECM, leading to a myofibroblast phenotype with enhanced matrix deposition and contractility. This process is accompanied by an altered miRNA expression pattern. miRNAs, prominently downregulated (blue arrow), and miRNAs shown to be upregulated (red arrow) during myofibroblastic activation are indicated.

Whereas the antifibrotic miRNAs, such as the members of the miR-29 family, miR-19, miR-150 and miR-133, repressing the myofibroblastic features, such as collagen synthesis or smooth muscle actin (SMA) synthesis, decrease after myofibroblastic transdifferentiation [25,43,63,64,65], others, such as miR-221/222, the neuronal miRNAs, miR-9, miR-125b, miR-128 or the miR-214, are increased [46,61,66]. Particularly, miR-221/222 expression is increased in cultivated primary HSC upon induction by the NF-kb activator, which is suggested to be a potential biomarker of stellate cell activation and linked to increased *Col1A1* expression during fibrosis [46]. Interestingly, miR-214 upregulated in activated HSC was shown to be controlled by the master transcription factor, Twist-1, [66]. miR-214 has to be considered as a main therapeutic target, because genetically or therapeutically silencing resulted in highly efficient inhibition of renal fibrosis [67]. Recent data of Noetel *et al.* could demonstrate a prominent upregulation of the neuronal miRNAs, miR-9, miR-125, and miR-128 in HSC upon myofibroblastic activation. These miRNAs were suggested to target several members of the chemokine and the chemokine receptor family [61].

**Figure 2:**
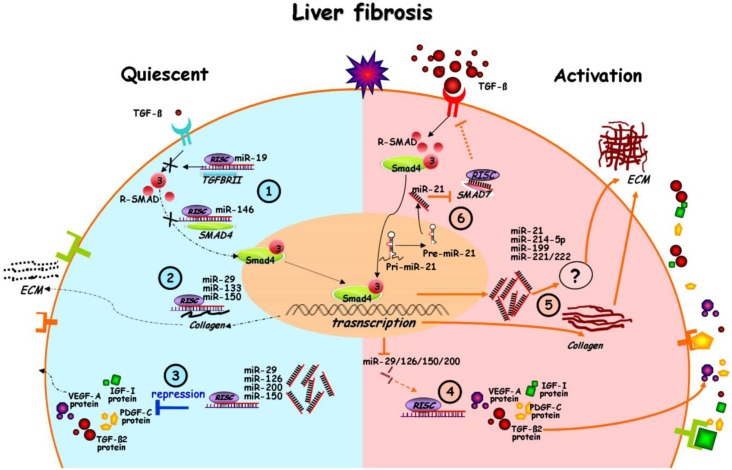
miRNAs in the interplay of signaling in quiescent and activated HSCs after chronic liver injury.

Lakner *et al.* identified 55 miRNAs that are divergently expressed in quiescent *versus* activated HSCs by microarray analyses. Hereby, miR-19b was proven to repress TGF-β signaling by targeting *TGF-β receptor type II* expression, which, in turn, resulted in a decrease of TGF-β signaling followed by reduced expression of collagen subunits (*Col1A1* and *ColA2*) [25]. Furthermore, the miR-29 and miR-21 function is closely linked to the profibrogenic TGF-β pathway [27,65,68,69]. Hence, miRNAs definitely contribute to the profibrogenic changes during fibrogenesis, not only by targeting ECM production, but also by interaction with the TGF-β signaling pathway (Figure 2).

## 4. miRNA in the Interplay of Profibrogenic Pathways

miR-29 expression is strongly regulated by TGF-β, as well as oppositionally by the TGF-β antagonist, hepatocyte growth factor (HGF). Whereas TGF-β inhibits epithelial proliferation, HGF exerts high mitogenic potential on epithelial cells and strong antifibrogenic functions on fibroblasts. Thus, the studies of Kwiecinski *et al.* revealed that HGF mediates antifibrogenic effects by induction and restoring of miR-29 expression, which, in turn, is followed by a significant decrease of collagen I and collagen IV subtypes [64]. However, miR-29 is not only regulated by the interplay of TGF-β and HGF, but also by other factors involved in inflammation and fibrosis, like PDGF-BB [65] and interferon-α [70].

In addition to its role in ECM repression, miR-29 regulates the growth factor profile of HSC by targeting profibrogenic mediators, such as IGF-I and PDGF-C [71]. Moreover, Sekyia *et al.* collected evidence that also the PDGF-β receptor is targeted by miR-29 regulation [65]. This is of special interest, because miR-29 itself is downregulated in HSC after PDGF-BB stimulation [65].

Additionally to miR-29, miR-146 is also decreased by TGF-β. TGF-β mediates its profibrogenic functions mainly by the smad pathway. In particular, after ligand binding and TGF-β receptor I phosphorylation, smad-3 activation and its interaction with smad-4 is crucial for HSC activation [58]. After smad-3/-4 transduction into the nucleus, the smad complexes are involved in the transcriptional control of a wide range of genes. While miR-146 is repressed by TGF-β signals, it suppresses TGF-β signals by targeting *smad-4* expression. Furthermore, TGF-β signaling is also inhibited by the antifibrotic microRNAs, miR-150 and miR-200a, which, in addition to their function in ECM regulation, target the expression of the signal transducer, smad-3, as well as of TGF-β2 [47,63] (Figure 2).

In quiescent HSCs, antifibrotic miRNAs, like miR-19, miR-146, miR-29, miR-133, miR-150, miR-126 and miR-200, are highly expressed. High levels of these miRNAs, in turn, lead to reduced synthesis of the TGF-β receptor II or smad-4 (1) of ECM, mainly the collagen subunits (2); or synthesis of profibrogenic mediators, e.g., VEGF-A, TGF-β2, IGF-I, PDGF-C (3); After chronic liver injury, HSCs get activated and transduce into a myofibroblastic phenotype. Myofibroblastic transition and autocrine TGF-β stimulation changes the miRNA profile, as shown in Figure 1. In response to TGF-β exposure, miR-29, miR-126, miR-150 and miR-200 are downregulated, resulting in the abolished repression of profibrogenic mediators (4); The reduced miR-29/133/150 levels then contribute to enhanced synthesis and deposition of ECM. Whereas some miRNAs are reduced by TGF-β, others, such as miR-21/214-5p/199/221/222, are stimulated and elevate ECM synthesis via an unknown pathway (5); TGF-β induced miR-21 expression results in a decrease of the TGF-β inhibitory smad-7 protein (6) (Dashed arrow lines indicate the suppression of pathways and gene expression, whereas solid arrow lines indicate stimulation).

miR-126 is closely linked to the vascular endothelial growth factor (VEGF) signaling, which is a crucial in angiogenesis, but affects fibrosis by the induction of HSC proliferation and ECM synthesis. During myofibroblastic HSC transition, miR-126 is lost, causing an increased synthesis of ECM proteins, but also of VEGF-A [72,73] (Figure 2).

Whereas the antifibrotic miRNAs are repressed by TGF-β, others, such as miR-214-5p and miR-21, are induced [74] in agreement with their enrichment during liver fibrosis [66,75]. Especially miR-21 was shown to be positively regulated in cancer cells by the TGF-β/smad pathway on both, the transcriptional and the miRNA processing level [68]. miR-21 further triggers the TGF-β/smad pathway by targeting the inhibitory smad protein, smad-7 (Figure 2). Furthermore, in stellate cells, the profibrogenic function of miR-21 is suggested to be also mediated by Pten inhibition, which, in turn, leads to Akt activation [76]. In conclusion, the altered expression of a wide panel of miRNAs affects fibrosis progression by its inhibition of ECM synthesis and its influence on central signaling pathways in HSC.

## 5. Perspectives

The high impact of miRNAs on the progression of fibrosis by the inhibition of ECM or by interfering with profibrogenic pathways announces the promising potential of miRNAs as biomarkers and targets of novel antifibrotic therapeutic strategies. Although, presently, miRNAs are not yet used as diagnostic biomarkers for fibrosis, in liver cancer, the diagnostic potential of miRNAs is already demonstrated by the low levels of miR-26, indicating the successful response to interferon-a therapy [77]. However, since miRNAs are released into the blood stream, circulating miRNA levels might serve even more efficiently as indicators of fibrosis [78,79]. Furthermore, the important function of many miRNAs acting as anti- or pro-fibrogenic factors emphasizes their role as therapeutic targets. The successful inhibition of miR-122 by antagonizing oligonucleotides [35] has successfully illustrated the proof of principle of this novel therapeutic strategy. However, whereas drug delivery to liver parenchyma is highly efficient, organ and cell-type specific miRNA targeting has to be achieved in liver fibrosis. Thus, in order to avoid cell unspecific side effects of antifibrotic therapies, that target miRNAs involved in liver fibrosis, an HSC- or myofibroblast specific approach of drug delivery is needed.
